# The Value of Food Allergy Prevention in Clinical Practice in Pediatrics: Targeting Early Life

**DOI:** 10.3390/children5020014

**Published:** 2018-01-23

**Authors:** Katherine Anagnostou, Jordan Scott Orange

**Affiliations:** 1 Section of Immunology, Allergy and Rheumatology, Department of Pediatrics, Baylor College of Medicine, Feigin Center, Houston, TX 77030, USA; orange@bcm.edu; 2 Section of Immunology, Allergy and Rheumatology, Department of Pediatrics, Texas Children’s Hospital, Houston, TX 77030, USA

**Keywords:** food allergy, prevention, children, primary care

## Abstract

Food allergies are common and increasing in prevalence, representing a major health concern in many countries around the world. In an effort to diminish the burden of food allergy, many research studies have focused on prevention, and recent findings have revolutionized the way we introduce allergenic foods in early life. We discuss the role of early allergenic food introduction and the value of food allergy prevention in this manuscript.

Food allergies are common and increasing in prevalence, representing a major health concern in many countries around the world. In the United States, approximately 4–8% of children are affected, with the prevalence of food allergy being highest in infants and toddlers, the ‘eight major allergenic foods’ in childhood being milk, egg, peanut, tree nuts, shellfish, fish, wheat and soy [1]. Food allergies pose a significant burden on the affected patients and their families [2]. Dietary restrictions, high levels of anxiety and social limitations are common problems resulting in decreased quality of life. At the same time, this is an area of intense research interest that constantly unveils new findings. As a result, our knowledge on food allergies steadily increases, guidelines change and new prevention and management approaches emerge in this rapidly-evolving field.

Taking new research evidence into account and considering the many controversies and unknowns that still exist in the food allergy field, we believe there is utility to providing a separate food allergy prevention consultation to infants/families at risk. This could also, in time, develop into dedicated food allergy prevention services, depending on population needs and healthcare resources’ availability with a strong possibility for telehealth components. The aim would be to offer advice and support to at-risk families, provide focused clinical and dietary interventions to infants and young children, educate parents and raise awareness with regards to food allergy prevention strategies, including new research findings, as they emerge. Infants at risk of peanut allergy would include those with early onset or severe eczema and/or egg allergy, as stated in recently-published national guidelines [3]. Infants at risk of food allergy in general may include those with existing food allergies, other atopic conditions (such as eczema, allergic rhinitis, asthma) or strong family history of atopy [4,5], although there is a clear need for additional research to clarify risk factors further. 

Recent studies have revolutionized the way we manage food allergies, especially in early life. The majority of recent studies has focused on early allergenic food introduction, but nutritional supplements and the role of microbiome have also been areas of intense research interest [6,7,8]. There have been significant changes in our approach to allergenic food introduction, as we now have evidence that early peanut introduction into the diet of high-risk infants, between 4 and 11 months of age, has the potential to prevent the subsequent development of peanut allergy [9]. This new evidence has resulted in a dramatic change to our feeding guidelines for infants, with the National Institute of Allergy and Infectious Diseases (NIAID) publishing new consensus guidelines on early peanut introduction in January 2017 [3]. Most interestingly, it has been highlighted that consumption of a minimum dose (2 g of peanut), at least three times per week may be required in order for this intervention to be successful. Several research trials have also investigated early egg introduction (both raw and cooked) with results appearing safer for introduction of lower amounts of egg protein, in a less allergenic form [10,11,12,13]. Studies have not been limited to at-risk populations; in fact, the Enquiring About Tolerance (EAT) study reported on over 1300 infants from the general population, comparing early weaning (between three and six months of age) to allergenic foods versus weaning after six months of age, alongside regular breastfeeding in both groups. Despite the intention-to-treat analysis not showing any efficacy with early allergenic food introduction, the per-protocol analysis (infants compliant with the dietary study requirements) clearly revealed a significantly lower prevalence of any food allergy in the early introduction group [14]. Considering that a large number of participants in the early introduction group were excluded due to poor compliance with the study protocol, results must be interpreted with caution.

The findings of these research studies have presented multiple marketing opportunities, with many new products offered to parents, in an effort to achieve the goal of early introduction. Recently, the Food and Drug Administration (FDA) has announced that in response to a request from manufacturers, it will allow a ‘qualified health claim’ on food labels containing ground peanut that coincides with the NIAID recommendations. The claim reads as follows: “For most infants with severe eczema and/or egg allergy who are already eating solid foods, introducing foods containing ground peanuts between 4 and 10 months of age and continuing consumption may reduce the risk of developing peanut allergy by 5 years of age. FDA has determined, however, that the evidence supporting this claim is limited to one study. If your infant has severe eczema and/or egg allergy, check with your infant’s healthcare provider before feeding foods containing ground peanuts” [15]. This field and the claim itself can be confusing for families to navigate; faced with a plethora of consumer commercial offerings, expert advice is needed, especially since certain products do not clearly address the issue of dose and frequency required to achieve the desired benefit and may also not conform to the recent NIAID guidelines.

Infants at risk of food allergy development present a number of challenges in pediatric allergy. Firstly, early onset and severe eczema have been associated with an increase in the risk of food allergy development [16]. However, the diagnosis, severity assessment and management of eczema is not always consistent and may vary amongst different healthcare providers and families. Secondly, infants are often tested with specific immunoglobulin E (IgE) to a variety of food allergens (also known as ‘food allergy panels’), without a clear indication, resulting in many false positive results and unnecessary dietary restrictions. Food challenges considered the gold standard for diagnosis are rarely performed outside specialist allergy services as they are time consuming and also carry a risk of severe allergic reactions. Thirdly, parents are frequently confused with regards to varying advice on allergenic food introduction, the role of breastfeeding, the use of hydrolyzed formulas and the role of probiotics in food allergy prevention. Finally, infants may not be referred to the allergy clinic until they are much older, due to misconceptions regarding early use of testing modalities and the need for further investigation. For instance, families report in our allergy clinic that they were unaware skin testing could be performed at such an early age or were told that the child was ‘too young’ for any beneficial allergy intervention. A dedicated clinician and dietician would help address all issues mentioned above, based on the latest available evidence. An important consideration would be the inclusion of pregnant or recently postpartum mothers in the clinic consultations. It is challenging to imagine how this complex topic might be integrated into the already robustly dedicated early life well-child visits of a pediatrician or family physician. That said, we appreciate that a solution is needed through active research and experience to be able to integrate guidance effectively into routine age-specific pediatric visits.

It is important to highlight that this pediatric age group may represent an ideal target population for food allergy interventions, from various aspects. First, the immune system in infancy is still in development and therefore likely more malleable to interventions that aim to achieve permanent changes. Second, it has been speculated that a ‘window of opportunity’ may exist for food allergy prevention with early introduction of allergenic foods before the first year of life. Third, early skin care with regular moisturizing has been shown to prevent eczema development and potentially disrupt the subsequent development of food allergies and other allergic-related conditions (a process also known as the ‘allergic march’) [17].

Key benefits for the selected families receiving a food allergy prevention consult would include timely access to a clinic with current expert clinical input, the opportunity to discuss allergenic food introduction, suitability of commercially available products and other diet-related issues in infancy (especially in light of the clinical characteristics of the infant), avoidance of unnecessary dietary exclusions, which, in addition to nutritional deficiencies, could also increase the risk of food allergy development, early assessment of food allergy risk and potential for interventions that prevent food allergy development. The role of primary care physicians (PCPs) in identifying families at risk, offering early-life advice and disseminating current research information about allergenic food introduction is crucial. PCP education therefore is an important step in achieving the goal of food allergy prevention. For complex patients or families seeking additional advice, an early referral to an allergist would also be appropriate and should be encouraged.

We conclude that there is considerable value presently in a food allergy prevention consultation for the at-risk population, with a focus on early life interventions. The service may be provided by PCPs well educated on the subject of allergenic food introduction and allergy specialists for more complex cases. This approach will not only provide education and support for families with at-risk infants and young children, but at the same time, also promote and raise awareness of new research findings, communicate changing guidelines and encourage the adoption of new evidence-based prevention approaches by the wider pediatric community, both parents and health professionals. It is clear that a strong collaboration between food allergy experts and the pediatric community is needed to navigate this period of rapid change and uncertainty to ensure the protection and betterment of infants facing the possibility of food allergy. Finally, the health economics of this model are largely unknown at present, and this area will require significant research.
